# An Unsolved Mystery

**Published:** 1920-07-17

**Authors:** 


### AN UNSOLVED MYSTERY.

There is considerable interest to be found in the further report on the great influenza epidemic just issued by the Registrar-General, but it will not come as a surprise to those who have followed these matters closely that the newest publication throws relatively little fresh light upon the influenza mystery.

When we read, however, the revised mortality figures and at the same time realise that the deaths actually attributed to influenza probably do not represent the total of those which it caused, the tremendous virulence of the infection is amazing. During all the outbreaks, entries under other headings, especially those of respiratory disease, were always bound to increase; but, even without these, the *deaths* of civilians corresponded to a mortality of 3,129 per 100,000 of the population. As the report explains, no similar mortality, since registration began, has ever been recorded, except in the case of the cholera outbreaks as long ago as 1849 when the corresponding number of deaths was 3,033. None of the previous influenza outbreaks can compare in virulence with that of 1918-19. During forty-six weeks—namely, June 23-May 10—the total deaths allocated to the disease were 151,446!

Also there was a change more remarkable than direct increase in mortality and that in the age incidence of the disease. This alteration was so extraordinarily sudden and so unexpected that it lead to no little diagnostic confusion. Formerly we have always considered influenza as far more dangerous when affecting those in the later years of life. In 1918-19 this situation was suddenly and inexplicably reversed. Those under the age of thirty-five died in appalling numbers, whereas those over fifty were largely immune. The authors of the report can find no parallel to this in the history of disease, and yet, as they say, the weight of medical testimony goes to show that the influenza of 1918 was essentially the same as that of former years. Even if attempt be made to explain the change by altered circumstances in the population, such as the aggregation of young women in munition factories or by any other of the war-time industrial and military expedients, it is found that no simple solution can possibly be given. To quote the only conclusion provided, we note simply that, " The alteration in age incidence accompanying the increased prevalence and fatality of the disease in 1918, seems to be more easily explained by a sudden change in the infecting organism than in the soil provided for its growth."

But if this be true it gives the medical profession considerable cause for reflection. We have the most fervent hope that the social and economic conditions of 1918 will not be from any cause repeated, and had we been able with reasonable certainty to lay the blame on the war and its effects for the terrible ravages of influenza, then we might have felt a reasonable security against its recurrence. If, on the other hand, we accept the explanation that the change rested with the infecting organism itself, it is not a happy thought that any one of our common and milder bacterial diseases may thus take on a sudden and disastrously plague-like form. It is true that some have claimed a " new " organism as the cause of influenza, but most authorities will, we believe, be in agreement with the report in its suggestion that the specific type of infection remained essentially the same. Only the virulence was amazingly increased—and the age incidence absolutely reversed.

Another fact is noticeable also. Neutral countries, and some belligerent countries indeed, where none of the privations suffered by Europe occurred, were found to be affected just as severely and to show exactly the same curious anomalies when attacked. There has been an enormous amount of observational work done, volumes of literature published, but still we are far from solving the real mysteries of the past influenza outbreak.

